# Climate of Madras

**Published:** 1846-03

**Authors:** 


*Climate of Madras.*—During the months of January and February, the weather at Madras is cool and pleasant; and this period is considered to be the most healthy season of the year. The mean temperature of these months is 76° F.; the wind blows steadily from N. E. and E., and the average fall of rain is 1 inch 25 cents. In March, April, and part of May, the south (or as it is called, 'the along-shore wind') prevails, and is reckoned very unwholesome, particularly to old residents, who generally suffer during this time of the year from rheumatic pains; the mean temperature of these months is 85°, and the average of rain 1 inch 85 cents. In the early part of May, very violent gales of wind have occasionally been experienced, accompanied with heavy falls of rain; about the middle of the month, the hot land-wind commences, and blows generally with great violence from about midnight till 12 or 1 in the day, when it is succeeded by the sea-breeze, which at this season is very refreshing; the land-wind continues throughout June and July, the mean temperature of these months being 88°, and the average of rain 2 inches

20 cents. In the beginning of July there are generally heavy showers of rain, which diminish the heat of the land-wind ; but it continues to blow during the month, though with less violence: mean temperature 85°, average fall of rain 3.37. In the month of August and September the weather becomes cloudy, close, and oppressive ; the sea-breeze being uncertain, and the winds generally light and variable, with frequent calms ; heavy falls of rain, ushered in by thunder and lightning, also occur in these months, that the cholera has generally raged epidemically at Madras. About the beginning of October the N. E. monsoon commences, and continues through the months of November and December ; in October heavy gales of wind are very frequently experienced : the weather is cool and damp, the mean temperature 80°, and the average of rain 30 inches.

*Average Medium Temperature throughout the Year, for* 10 *Years.*

| 1829 | 1830 | 1831 | 1832 | 1833 | 1834 | 1835 | 1836 | 1837 | 1838 |
|---|---|---|---|---|---|---|---|---|---|
| Fahrenheit. 3 | 81 | 81½ | 84 | 83 | 87 | 82 | 77½ | 82½ | 86½ |

*Monthly Journal of Med. Science.*

